# Supplementary material for: Repat33 Acts as a Downstream Component of Eicosanoid Signaling Pathway Mediating Immune Responses of Spodoptera exigua, a Lepidopteran Insect
Source: Insects. 2021 May 14;12(5):449. doi: 10.3390/insects12050449 (PMC8156158; doi:10.3390/insects12050449)
Supplement: Supplementary file 1 [file insects-12-00449-s001.zip › insects-1205451-supplementary.pdf]

Table S1. List of primers used in this study

Genes	Orientation	Sequence (5′ - 3′)	Uses	Annealing temperature (ºC)	Expected size (bp)
Repat33	Forward	GTCTCTGTGGACACATGTTACT	RT-PCR	52.0	297
	Reverse	CCCTTTCCCTTCTCACTCTTC	RT-qPCR		
T7+Repat33	Forward	TAATACGACTCACTATAGGGAGA GTCTCTGTGGACACATGTTACT	RNAi	65.0	343
	Reverse	TAATACGACTCACTATAGGGAGA CCCTTTCCCTTCTCACTCTTC			
Repat46	Forward	GTTGTGTCGCAGTTGGCTAC	RT-PCR	54.4	167
	Reverse	TACCTCTTGCGTTTGCACCA	RT-qPCR		
Repat20	Forward	AAAACTCCCCCACTGGAACG	RT-PCR	54.4	220
	Reverse	CTACCGCCTAGGGTAGGGTT	RT-qPCR		
Repat12	Forward	CAGTCTGCTATGATGCCGCT	RT-PCR	54.4	293
	Reverse	CTTCCGCAGTGGAGAACGAA	RT-qPCR		
Repat16	Forward	AGCCTGCTATGGAAGTGCTG	RT-PCR	54.4	227
	Reverse	TGCCGGGAGATCCCTCATAA	RT-qPCR		
Repat30	Forward	CGTTTTCAAAGCCGACGAGG	RT-PCR	54.4	212
	Reverse	CCCTTTGGCTCTTGAGCTTG	RT-qPCR		
A ribosomal protein RL32	Forward	ATGCCCAACATTGGTTACGG	RT-PCR	52.0	240
	Reverse	TTCGTTCTCCTGGCTGCGGA	RT-qPCR		
Apolipophorin III	Forward	AGTGTCGCCAAGTTGTTCGTG	RT-qPCR	52.0	420
	Reverse	CTCCTGCGCGGTGTTCTGCA			
Attacin 1	Forward	GCTTTCCTCTCCAGGAATATG	RT-qPCR	52.0	276
	Reverse	CCTTAGAGTAAATCCAGTGG			
Attacin 2	Forward	TCCCGAATGTGCCCAACTTC	RT-qPCR	52.0	254
	Reverse	GAAAGATCTGCCGAAAGTAAG			
Defensin	Forward	ATGGGTGTTAAGGTAATAAATGTG	RT-qPCR	52.0	303
	Reverse	GCAACTACATGTATGACTAACGC			
Gallerimycin	Forward	TCAGTCATGAAAGCTTGCGTA	RT-qPCR	52.0	222
	Reverse	TCGCACACATTGGCATCCATTC			
Gloverin	Forward	CGTGGACATCTTCAGGGCC	RT-qPCR	52.0	277
	Reverse	GTCGTGTTCAATGCCACC			
Lysozyme	Forward	ATGCAAAAGCTAACGGTTTTC	RT-qPCR	52.0	385
	Reverse	GATTCTTCCATCCATACCAG			
Transferrin 1	Forward	GTCCCTCTCTGTCCTGAAGG	RT-qPCR	52.0	370
	Reverse	CAGAAACACGAAGAAAGATGG			
Transferrin 2	Forward	GATGTTCTGGCGCAGCTGTC	RT-qPCR	52.0	288
	Reverse	CCGGCTGAACGCAAACACAG			
Cecropin	Forward	ATCGTTTAGCTTCGTGTTCGC	RT-qPCR	52.0	251
	Reverse	CTTTCTTTTACCACACGGTTG			
